# Can vitamin D status influence seroconversion to SARS-COV2 vaccines?

**DOI:** 10.3389/fimmu.2022.1038316

**Published:** 2022-12-19

**Authors:** Endrit Shahini, Francesco Pesce, Antonella Argentiero, Antonio Giovanni Solimando

**Affiliations:** ^1^ Gastroenterology Unit, National Institute of Gastroenterology S. De Bellis Research Hospital (IRCCS), Castellana Grotte, Italy; ^2^ Nephrology, Dialysis and Transplantation Unit, Department of Precision and Regenerative Medicine and Ionian Area - (DiMePRe-J), University of Bari “A. Moro”, Bari, Italy; ^3^ Medical Oncology Unit, IRCCS Istituto Tumori “Giovanni Paolo II” of Bari, Bari, Italy; ^4^ Guido Baccelli Unit of Internal Medicine, Department of Precision and Regenerative Medicine and Ionian Area - (DiMePRe-J), University of Bari “A. Moro”, Bari, Italy

**Keywords:** COVID-19, coronavirus, vitamin D, antibodies, serology, autoimmune disorders, pneumonia

## Abstract

Existing data indicate an association between vitamin D deficiency and increased severity of respiratory distress due to COVID-19 infection, especially in high-risk populations. To date, the effect of vitamin D on immunogenicity to SARS-CoV-2 vaccines has been investigated solely in young healthcare workers in a few studies, yielding conflicting findings, yet highlighting that the response to immunization is inversely related to age. Vitamin D status can potentially influence the antibody titers in people with a previous (or naïve) SARS-CoV-2 infection and vaccination, given its role in immune regulatory functions. From this standpoint, vitamin D supplementation can help reduce the risk of SARS-CoV-2 infection, COVID-19 severity/mortality and rebalance immunological function, particularly in subjects with vigorous T lymphocyte responses to COVID-19. However, more research is needed to establish a correlation between vitamin D status and the generation of protective serological responses to SARS-CoV-2 vaccination.

## 1 Introduction

*Altmann D.M.* et al., in their comment, have elaborated elegantly on the plethora of unresolved issues with the reliability of serological tests used for severe acute respiratory syndrome coronavirus 2 (SARS-CoV-2) identification, after coronavirus disease 2019 (COVID-19) resolution (1). U.S. has been widely tested for SARS-CoV-2 infection from March to April 27, 2020 resulting in 5.44 million people infected, followed by Italy (1.76 million). The identification of infected individuals relies on polymerase chain reaction (PCR) based tests for SARS-CoV-2. In contrast, antibody blood tests determine whether a past infection has occurred by detecting serum antibodies against the virus (1). Despite the higher sensitivity of IgA enzyme-linked immunosorbent assay (ELISA) in detecting antibody titers, their false-negative rates due to lower specificity suggest a particular caution when interpreting the results (2).

It has been observed that COVID-19 outbreaks, and mortality, in particular, have a higher prevalence in the Northern hemisphere, exhibiting a gradient according to the geographical distribution of hypovitaminosis D (3). This is mainly mediated by distinct Earth’s seasonal ultraviolet (UV) exposure, although another reasonable explanation could rely on certain atmospheric phenomena (3, 4).

UV radiation strength, exposure time, and skin colour are all known to influence vitamin D biosynthesis (5). Optimal conditions for vitamin D biosynthesis may differ in each individual, and seasonal changes in sunlight exposure may also have an impact (6–9). Notably, previous research has also shown that vitamin D levels can drastically decrease from summer to winter in adult and young (apparently) healthy subjects of different ethnicities (10–15).

Additionally, several studies on the relationship between sunlight exposure and the global COVID-19 pandemic have been conducted (6–8), with solar UV showing a direct effect on SARS-CoV-2 inactivation (16, 17). Furthermore, COVID-19 transmission in South America was aided by cold, dry, and windless conditions (18). Even though no conclusive studies are available, seasonal variations in SARS-CoV-2 illness may be influenced in several cases by a lack of sun exposure, which may impair vitamin D status, especially in at-risk patients (19, 20).

## 2 Vitamin D immunomodulatory properties in the COVID-19 setting

Vitamin D has pleiotropic effects on the immune system (3, 21). Active vitamin D (1,25-dihydroxyvitamin D3 [1,25-(OH)2D3] is synthesized in renal tubules and acts as a steroid hormone, influencing the expression of hundreds of genes (22). It induces cathelicidins and defensins, which concurrently down-regulate viral replication and pro-inflammatory cytokines that lead to potential interstitial pneumonia and acute respiratory distress syndrome (ARDS) after the onset of a cytokine storm (3, 21). Besides, vitamin D modulates adaptive immunity by suppressing T Helper cell type-1 (TH1) responses and promotes the induction of T regulatory cells that counterbalance inflammatory responses (21). Vitamin D inhibits the expression of renin and, therefore also affects the renin-angiotensin system (RAS)/angiotensin-converting enzyme 2 (ACE2) signaling axis (3). Interestingly, vitamin D exerts propitious anti-thrombotic actions in tissues directly or indirectly involved in thrombosis pathophysiology (21).

*Chauss* and Colleagues have presented new evidence that severe COVID-19 may be caused, in some people, by the lack of the ability to resolve an exuberant type I immune response. They specifically implicated vitamin D receptor (VDR) signaling in this process (23), showing that when vitamin D levels are low, human CD4+ T cells express more type 1 (IFNG) and type 17 genes. In contrast, IL-6R and IL-10 levels are lower. Vitamin D induces STAT3, BACH2, and JUN to increase IL-6R (reinforcing STAT3 activation) and IL-10, which may be necessary for converting the pro-inflammatory TH1 cell-type to that crucial in resolving type 1 inflammation in the setting of severe COVID-19 (23). *Minton K* also proposed that the impaired transcriptional response to vitamin D in patients with severe COVID-19 could be due to vitamin D deficiency or dysregulation of complement-induced autoregulatory VDR signaling (24). Thus, vitamin D supplementation may modulate IL-6 towards a favorable profile in COVID-19.

## 3 Evidences linking vitamin D and COVID-19 outcomes

A 2021 meta-analysis of fifty-four observational studies including a total of 1,403,715 individuals, found that all patients with severe deficiency, and/or insufficiency of vitamin D present an increased risk of ARDS requiring admission to intensive care unit (ICU) or mortality due to COVID-19 and a higher susceptibility to SARS-CoV-2 infection and related hospitalization (25).

Current evidence supports a solid relationship between vitamin D deficiency and increased severity of ARDS associated with COVID-19, especially in high-risk populations for hypovitaminosis D (*e.g.*, elderly, Northern people, hospitalized, cardiovascular disease, gastrointestinal diseases, chronic kidney disease, diabetics, obese, impaired immune function, dark-skinned ethnicities, vegetarians or vegans) (3, 21–44). The studies listed in the **Supplementary Material** support the association between vitamin D levels and COVID-19 outcomes.

Several studies have investigated vitamin D deficiency in the context of COVID-19 severity/mortality based on specific evidence of a protective effect of vitamin D supplementation (400-1000 IU daily) against respiratory tract infections resulting from two rigorous and large meta-analyses of randomized controlled trials (RCTs), particularly in individuals with baseline 25-hydroxyvitamin D3 [25(OH)D3] levels < 25 nmol/L (44, 45).

## 4 Vitamin D status and COVID-19 immunization

Antibody titers, after recovery from COVID-19, indicate healing, but still, there is uncertainty about their neutralizing nature. The levels of such antibodies can be theoretically influenced also by vitamin D status in patients with previous SARS-CoV-2 infection given the immunomodulatory properties of vitamin D, which also include the inhibition of lymphocyte proliferation and immunoglobulin production in healthy conditions (46).

### 4.1 Seroconversion in immune-mediated diseases

A meta-analysis published in 2022 found that seroconversion rates after SARS-CoV-2 vaccination are lower in patients with immune-mediated inflammatory diseases. Specific therapies (anti-TNF, anti-integrin, anti-IL17, anti-IL6, anti-IL12/23) do not affect seroconversion rates, whereas others (anti-CD20, anti-CTLA-4) have a negative impact (47). Notably, there is an inverse relationship between vitamin D status and the development of several autoimmune diseases (48). It is known that vitamin D counteracts the suppressive effect of inflammatory cytokines on CTLA-4 expression and regulatory function (49). Furthermore, 1,25-(OH)2D3 has a critical positive regulation of CTLA4, an essential negative regulator in immune responses (50).

### 4.2 Vitamin D supplementation

The conundrum has been raised of whether vitamin D would be beneficial in reducing the severity of COVID-19 illness, its requirement for hospitalization, and the length of symptoms.

Vitamin D supplementation may reduce the risk of SARS-CoV-2 infection, COVID-19 severity, and mortality risk (3). It may also help rebalance immunological function in high-risk individuals, particularly those with COVID-19 who demonstrated strong T lymphocyte responses (1, 51).

To decrease the risk of infection and enhance the immunological reactivity against COVID-19, oral supplementation in patients with vitamin D deficiency has been initially suggested at a dosage of 10,000 IU/day of vitamin D3 for a few weeks, followed by a daily dosage of 5,000 IU (3, 52). The ideal target is a 25(OH)D3 over 40-60 ng/mL.

A meta-analysis of 6 RCTs involving 551 COVID-19 patients published in 2022 concluded that despite the heterogeneity of the included studies, vitamin D supplementation was beneficial in COVID-19 and was associated with a lower rate of ICU admission, mortality events, and RT-PCR positivity (53).

Based on a pilot study and several observational intervention studies in which the use of high doses of calcifediol dramatically reduced the need for ICU admission and the mortality rate, it was proposed a rapid correction of 25(OH)D3 deficiency in all patients in the early stages of COVID-19 (54). In particular, early administration of high-dose versus standard-dose vitamin D3 to at-risk older patients with COVID-19 improved the two-week mortality (55). Similarly, other *Authors* in a recent meta-analysis of thirteen observational and RCTs involving a total of 2,933 COVID-19 patients (56), concluded that high-dose cholecalciferol supplementation may be associated with better clinical outcomes, particularly when administered after the diagnosis of COVID-19.

On the contrary, another recent RCT found that a single high oral dose of vitamin D3 (200,000 IU) did not significantly reduce hospital length of stay in 237 COVID-19 patients hospitalized (57). Other previous RCTs produced mixed and inconsistent results, with no clear positive results, and two 2021 meta-analyses found no significant difference between vitamin D supplementation and major health outcomes in COVID-19 (58–61).

*Bergman P* discussed vitamin D’s role in protecting against COVID-19 infection in a recent Editorial (62). He hypothesized that the association could be due to reverse causality or confounding. However, because COVID-19 vaccination was being rolled out during the previous null studies, he did not rule out the possibility that the highly effective vaccination could have masked any vitamin D effect.

Therefore, questions about the correct dosage, period, and methods of administration of vitamin D still need to be answered. A group from Harvard Medical School is currently investigating this question in a new pragmatic, cluster-randomized, double-blinded trial officially named VIVID (Vitamin D for COVID Trial), which is experimenting with the effects of a higher dose of vitamin D above 3,000 IU per day on disease progression and post-exposure prophylaxis for COVID-19 infection (63).

## 5 Vitamin D and gastrointestinal diseases during the COVID-19 scenario

Notably, even though SARS-CoV-2 is a lung-tropic virus damaging the respiratory tract by binding to the ACE2 cell-surface compounds found on alveolar pulmonary epithelial cells, gastrointestinal symptoms are common in COVID-19 patients and often precede respiratory tract disease. Recently, it was discovered that SARS-CoV-2 actively replicates in the gut, particularly in mature enterocytes expressing the ACE2 viral receptor and the TMPRSS4 protease (64).

According to current research, the host’s gut microbiota is key in influencing their health, particularly as a mediator of chronic systemic low-grade inflammation (65). During SARS-CoV-2 infection, the viral balance in the gastrointestinal tract may be disrupted, influencing the equilibrium of the intestinal microbiota (66).

There is mounting evidence that a cross-talk between the gut microbiome, vitamin D, and the RAS/ACE2 system is key for the elderly immune system’s balanced functioning. It has been proposed that the state of the gut microbiome prior to infection determines the outcome of COVID-19 sepsis and may also be a critical factor in vaccination success. Evidence suggests a complex relationship between COVID-19 severity and gut microbiota, ACE-2 expression, and vitamin D. Vitamin D promotes the growth of commensal strains of Bifida and Firmicutes species in the gut (67).

Anyway, for those with low vitamin D levels below 50 nmol/L, the 1000-2000 IU/day supplementation may be appropriate. Importantly, high-risk individuals with gastrointestinal malabsorption diseases such as celiac disease, Crohn’s disease, intestinal bypass surgery, hepatobiliary/pancreatic diseases, and sarcopenia, should continue to receive vitamin D and calcium.

Taking vitamin D supplements could have multifaced advantages, including the fact that COVID-19 may act as a potential trigger factor for several autoimmune diseases, such as celiac disease, in predisposed patients (68, 69).

## 6 Vitamin D levels and immune response to SARS-CoV-2 vaccines

*Grifoni A* and *Sette A* found early that CD4+ T cell responses to spike protein, the main target of most vaccine efforts, were robust and correlated with the anti-SARS-CoV-2 IgG and IgA magnitude titers in COVID-19 convalescent patients. Notably, they also found a significant prevalence of SARS-CoV-2-reactive CD4+ T cells in uninfected people, implying cross-reactive T cells between circulating commonly coronaviruses and SARS-CoV-2 (70).

On the other hand, a 2022 Chinese multicenter study found that, when compared to seronegative healthy controls, patients with solid malignancies who failed the standard 2-dose inactivated COVID-19 vaccines had a relatively poor humoral response to the third dose of vaccines, which was associated with low vitamin D levels and intake (71).

Several studies have confirmed that vitamin D and its pathway polymorphism can improve vaccine efficacy for infectious diseases like influenza, measles, rubella, pneumococcal/meningococcal/human papillomavirus disease, tuberculosis, or hepatitis B in a variety of ways (72, 73). Only a few studies excluded that serum 25(OH)D3 concentrations affected the immunogenicity of influenza vaccination in the elderly (74, 75).

To date, the impact of vitamin D on immunogenicity to SARS-CoV-2 vaccines has been studied mainly in healthy healthcare workers (HCWs) in a few studies with contrasting results.

In a 2021 German observational trial, SARS-CoV-2 IgG and neutralization potency and 25(OH)D3 concentrations were measured in a cohort of 126 mostly female and young healthy HCWs 24 weeks after BNT162b2 vaccination. The antibody response was inversely related to age. A similar trend in neutralizing antibodies was observed, though significance was lost at the final sampling time point. Still, the dynamic change of SARS-CoV-2 IgG as a function of 25(OH)D3 status showed no significant differences (76). Nevertheless, the results could have been influenced by the fact that almost 50% of participants were supplementing vitamin D during winter, and the final blood sample was taken in summer when vitamin D levels usually increase even in those not supplementing. Also, the number of patients whose antibody level values were available at the end of the study was drastically reduced compared to the beginning to make reliable results (n = 56 vs. 126). Finally, the authors did not specify the proportion of vitamin D deficiency/insufficiency among participants.

Similarly, three sub-studies were conducted within the CORONAVIT RCT UK adults (n = 2,808) with low vitamin D levels to see if vitamin D supplements could improve the immunogenicity and efficacy of SARS-CoV-2 vaccination. Vitamin D supplementation did not affect the risk of SARS-CoV-2 infection in vaccinated participants when 800 IU/day or 3200 IU/day supplementation was used versus no supplementation, nor on combined IgG-IgA-IgM anti-Spike or neutralizing antibody titers. However, the study had some biases because 69.3% of the mostly white participants (with various comorbidities) received two doses of ChAdOx1nCoV-19, while the remaining participants received two doses of BNT162b2; nearly 5% of subjects had SARS-CoV-2 infection before vaccination and only 11% of patients had vitamin D deficiency, whereas vitamin D levels were not determined in the non-supplemented group (n = 908) (77).

Another Romanian study looked at antibody responses after BNT162b2 vaccination concerning previous SARS-CoV-2 infection status and age and looked for potential biomarkers associated with changes in immune responses. They discovered that prior infection yielded an 8-fold growth in antibody titers, an effect that was weaker in people over 60 years old and unaffected by vitamin D serum levels (78).

Conversely, in an Italian study, the *Authors* explored the relationship between serum 25(OH)D3 levels and the immune response evoked by the BNT162b2 vaccine in a group of 101 HCWs, the majority of whom were young females naïve for SARS-CoV-2 infection (79). There were significant correlations between baseline 25(OH)D3 concentration and anti-spike IgG response and neutralizing antibody titer 24 weeks after the second dose when serum 25(OH)D3 levels increased significantly. These findings may be justified by the coincidence of the summer season after the six months when sun exposure usually increases vitamin D levels. As a result, 25(OH)D3 levels at the time of vaccination may influence the persistence of the antibody response, even if further studies are required to corroborate these findings, particularly in vulnerable populations.

Interestingly, a recent Indonesian study followed 194 volunteers for eight months after receiving two inactivated SARS-CoV2 vaccination injections. The subjects with low vitamin D levels had lower IFN-γ and IL-12 levels 6 to 7 weeks after the second vaccine injection. Also, those with low IFN-γ levels had a higher risk of COVID-19 infections during follow-up. Therefore, inadequate vitamin D levels were associated with a lower Th1 immune response, whereas adequate IFN-γ levels were necessary to improve vaccine efficacy (80).

Similarly, a 2022 single-center study examined the effect of vitamin D on the response to SARS-CoV-2 immunization after the first BNT162b2 vaccine dose as measured by anti-SARS-CoV-2 Spike IgG concentration in 97 UK HCWs, primarily white and female, over 8 weeks. After two months, the response to immunization was inversely related to age and significantly affected by vitamin D status. Younger people with 25(OH)D3 levels greater than 50 nmol/L had a 29.3% higher IgG peak value. Antibody concentrations decreased in an age-dependent manner, with younger HCWs with higher peaks decreasing faster to comparable concentrations across ages at week 8 (81). These findings support the authors’ conclusion that a booster immunization program should be planned after sun exposure (end of summer or early autumn) or following vitamin D supplementation.

**Figure 1:**
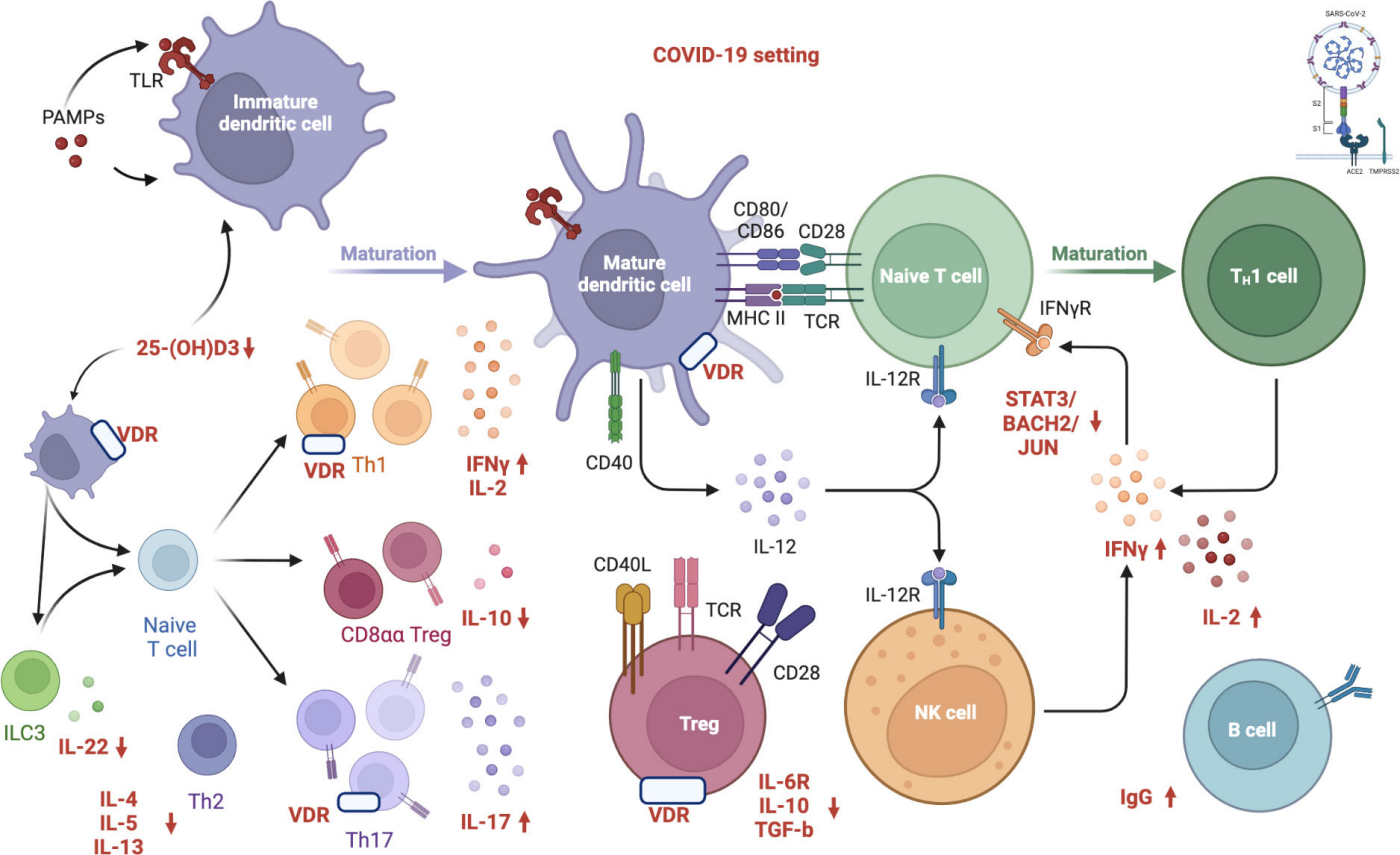
Proposed mechanism linking vitamin D deficiency to COVID-19 antibody response. “Created with BioRender.com” (Agreement number: MW24DBIWVD, Academic License). Vitamin D inhibits both the acquired immune system and stimulates the innate immune response as well. VDR modulates immunity processes, such as anti-microbial/anti-viral defense and the induction of T-cell tolerance. Several immune cells express the VDR and CYP27B1 (enzyme 1α-hydroxylase that converts vitamin D to the active form, 1,25-dihydroxyvitamin D3). The activation of innate immunity in SARS-CoV-2 infection seems to result in an increased local 1,25(OH)2D3 production and viral clearance while altering the pro-inflammatory response. The active form of vitamin D, 1,25(OH)2D3, is an inhibitor of dendritic cell maturation and functions as an immune modulator, reducing the activation of the acquired immune system. Furthermore, vitamin D induces transcription factors, including STAT3, BACH2, and JUN, which repress TH1 and TH17 responses and increase IL-10 via IL-6-STAT3 signaling, which is crucial in resolving type 1 inflammation in severe COVID-19 patients. Low vitamin D levels impair innate immune function, inactivating T reg cells and expanding the activated T and B cells, along with the immunoglobulin production. Specifically, human CD4+ T cell activation is associated with high levels of IFNγ, IL-2, IL-17, and low levels of IL-6R, TGF-β, and IL-10, resulting in the pro-inflammatory TH1 skewing and potentially severe tissue damage. COVID-19: Coronavirus Disease 2019; SARS CoV 2: Severe Acute Respiratory Syndrome Coronavirus 2; S1: S1 domain of the coronavirus spike (S) protein; TMPRSS2: Transmembrane protease serine 2; S2: S2 domain of the coronavirus spike (S) protein; ACE2: Angiotensin-Converting Enzyme 2; PAMP: Pathogen Associated Molecular Patterns; TLR: Toll-like receptor; 25(OH)D3: 25-hydroxycholecalciferol; ILC3: Type 3 Innate Lymphoid Cells; IL-22: Interleukin 22; IL-4: Interleukin 4; IL-5: Interleukin 5; IL-13: Interleukin 13; Th2: Type 2 T Helper Cell; Th1: Type 1 T Helper Cell; Th17: Type 17 T Helper Cell; VDR: Vitamin D Receptor; CD8αα: Cluster of Differentiation 8αα; Treg: Regulatory T cells; IFNγ: Gamma Interferon; IL-2: Interleukin 2; IL-10: Interleukin 10; IL-17: Interleukin 17; CD40: Cluster of Differentiation 40; CD80: Cluster of Differentiation 80; CD86: Cluster of Differentiation 86; CD28: Cluster of Differentiation 28; MHCII: Major Histocompatibility Complex; TCR: Toll-like receptor; IL-12: Interleukin 12; IL-12R: Interleukin-12 Receptor; CD40L: Cluster of Differentiation 40 Ligand; CD28: Cluster of Differentiation 28; IL-6R: Interleukin-6 Receptor; TGF-β: Transforming Growth Factor Beta; NK: Natural Killer; IFNγR: Gamma Interferon Receptor; STAT3: Signal Transducer And Activator Of Transcription 3; BACH2: BTB Domain And CNC Homolog 2; JUN: Jun proto-oncogene; IgG: Immunoglobulin G. APC: Antigen-presenting cells; VDR: vitamin D receptor.

**Figure 2:**
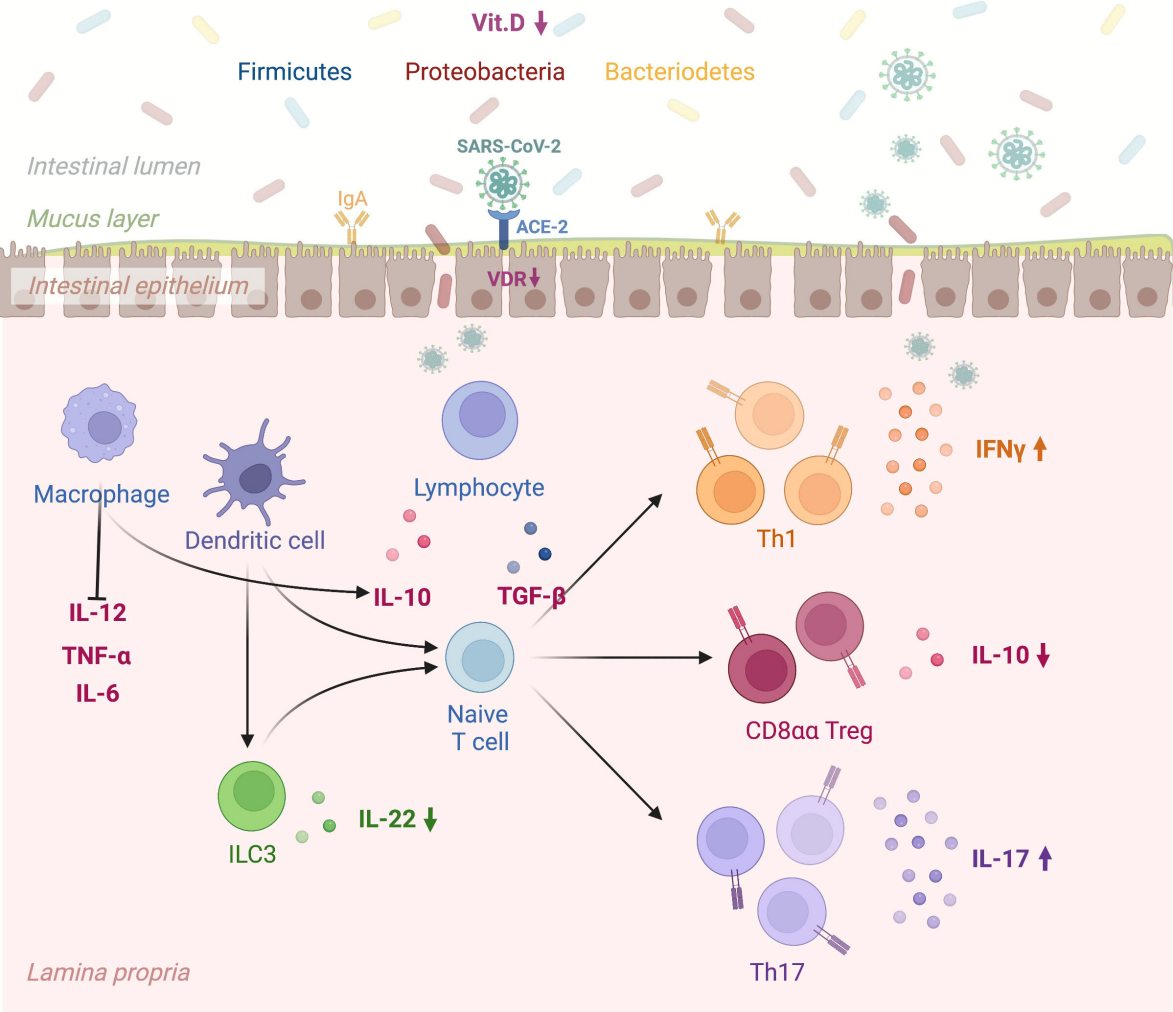
A schematic view linking vitamin D deficiency, the gut microbiome, and the intestinal immune response to COVID-19. “Created with BioRender.com” (Agreement number: ON24KBPV47, Academic License). Vitamin D promotes the expression of gap junction proteins within the gastrointestinal tract, which maintain barrier integrity and thus prevent tissue entry by bacteria from the gut microbiome. The VDR-vitamin D endocrine system can modulate acquired and innate immune system functions in viral infections. Specifically, SARS-COV-2 infection of the intestinal epithelium results in rapid viral replication. In the presence of vitamin D deficiency intestinal dendritic cells activated by viruses migrate to lymph nodes, where they earn an enhanced capacity to present antigen or T cell activation (e.g., CD4, CD8, TH, T reg, and ILC). Lower Vitamin D levels alter immune balance promoting pro-inflammatory cytokines and T Helper cell type-1 (TH1), 17 (TH17) and suppressing T regulatory response. Vit.D: Vitamin D; SARS CoV 2: Severe Acute Respiratory Syndrome Coronavirus 2; ACE-2: Angiotensin-Converting Enzyme 2; VDR: Vitamin D Receptor; IgA: Immunoglobulin A; IL-12: Interleukin 12; TNF-α: Transforming Growth Factor; IL-6: Interleukin-6; ILC3: Type 3 Innate Lymphoid Cells; IL-22: Interleukin 22; IL-10: Interleukin 10; TGF-β: Transforming Growth Factor Beta; Th1: Type 1 T Helper Cell; CD8αα T reg: Cluster of Differentiation 8αα T Regulatory Cell; Th17: Type 17 T Helper Cell; IFNγ: Gamma Interferon; IL-17: Interleukin 17. VDR: vitamin D receptor.

However, conclusive data associating antibody titers against the virus with vitamin D serum levels in COVID-19 patients still need to be included. **Figure 1** depicts in detail a proposed mechanism linking vitamin D deficiency to COVID-19 antibody response while **Figure 2** illustrates the role of vitamin D deficiency in the context of gut microbiome and the intestinal immune response concerning COVID-19.

Furthermore, as the next Gordian knot, we need to establish whether the host residual immunological activation after SARS-CoV-2 eradication will latently progress into full-blown B‐cell polyclonal or monoclonal expansion. At the state of our knowledge, this enigma is currently unsolvable as it requires long-term surveillance.

## 7 Conclusion

Further research is needed to determine a link between vitamin D status and the generation of protective serological responses to SARS-CoV-2 vaccination.

## Author contributions

Study conceptualization, ES. Drafting the original manuscript, ES. Supervision, FP, AA, and AS. Data collecting and curation, ES. Data gathering, manipulation, analysis, ES, FP, AA, and AS. All authors contributed to the article and approved the submitted version.
