# Corrigendum: Can vitamin D status influence seroconversion to SARS-COV2 vaccines?

**DOI:** 10.3389/fimmu.2023.1210147

**Published:** 2023-05-03

**Authors:** Endrit Shahini, Francesco Pesce, Antonella Argentiero, Antonio Giovanni Solimando

**Affiliations:** ^1^ Gastroenterology Unit, National Institute of Gastroenterology S. De Bellis Research Hospital (IRCCS), Castellana Grotte, Italy; ^2^ Nephrology, Dialysis and Transplantation Unit, Department of Precision and Regenerative Medicine and Ionian Area - (DiMePRe-J), University of Bari “A. Moro”, Bari, Italy; ^3^ Medical Oncology Unit, IRCCS Istituto Tumori “Giovanni Paolo II” of Bari, Bari, Italy; ^4^ Guido Baccelli Unit of Internal Medicine, Department of Precision and Regenerative Medicine and Ionian Area - (DiMePRe-J), University of Bari “A. Moro”, Bari, Italy

**Keywords:** COVID-19, coronavirus, vitamin D, antibodies, serology, autoimmune disorders, pneumonia

In the published article, there was an error in affiliation 1. Instead of “National Institute of Research “Saverio De Bellis”, Castellana Grotte, Italy”, it should be “Gastroenterology Unit, National Institute of Gastroenterology S. De Bellis Research Hospital (IRCCS), Castellana Grotte, Italy”.

The authors apologize for this error and state that this does not change the scientific conclusions of the article in any way. The original article has been updated.

