# Supplementary material for: Can vitamin D status influence seroconversion to SARS-COV2 vaccines?
Source: Front Immunol. 2022 Dec 19;13:1038316. doi: 10.3389/fimmu.2022.1038316 (PMC9806423; doi:10.3389/fimmu.2022.1038316)
Supplement: Supplementary file 1 [file Table_1.pdf]

Supplementary Material:

Supplementary Table 1. Studies that support the link between vitamin D levels and COVID-19 outcomes.

Author	Study type	Population (N pts.)	Serum Vit.D levels (cut-off, ng/mL)	Association Vit.D and COVID-19
D'Avolio A (2020)[27]	Retrospective	1,377	Median Vit.D value: 11	SARS-CoV-2 positive patients have significantly lower Vit.D levels
Merzon E (2020)[28]	Prospective, population-based epidemiological study	782	Suboptimal or low Vit.D < 30	Low Vit.D levels are independent risk factor for COVID-19 infection and hospitalization
Meltzer DO (2020)[29]	Single-center, retrospective cohort study,	489	Vit.D deficiency 25(OH)D <20 [or 1,25(OH)D <18 pg/mL]	Vit.D deficiency is associated with increased COVID-19 risk
Kaufman HW (2020)[30]	Retrospective, observational	191,779	Vit.D deficiency <20	SARS-CoV-2 positivity is strongly and inversely associated with circulating Vit.D levels
Chiodini I (2021)[25]	Meta-Analysis (54 observational studies)	1,403,715	Vit.D insufficiency <75 nmol/L; deficiency <50 nmol/L; severe deficiency <25 nmol/L	Low Vit.D levels have an increased risk of ARDS, ICU admission, or mortality, and a higher susceptibility to SARS-CoV-2 infection and related hospitalization
Liu N (2021)[31]	Systematic review and meta-analysis (10 observational studies)	361,934	N.A.	Low Vit.D status may be linked to an increased risk of COVID-19 infection
Kazemi A (2021)[32]	Systematic review (39 retrospective, prospective, cross-sectional, case-control, and RCT studies)	4,492	Vit.D deficiency <10, or ≤12 or <20, or ≤30	Vit.D is significantly related to SARS-CoV-2 infection, COVID-19 severity, and mortality

Sulli A (2021)[33]	Case-control	65	Vit.D deficiency <20; insufficiency 21–29	In elderly COVID-19 patients, Vit.D deficiency is associated with more severe lung involvement, a longer disease duration, and an increased mortality risk
Szarpak L (2021)[34]	Systematic review and meta-analysis (13 prospective, retrospective studym case-control studies)	14,485	NA	Low Vit.D levels are significantly associated with COVID-19 infection risk
AlSafar H (2021)[35]	Observational study	464	NA	Vit.D deficiency is linked to COVID-19 severity and mortality
Demir M (2021)[36]	Retrospective cohort study	260	Group I (Vit.D 0-10), Group II (10-20), Group III (20-30), and Group IV (> 30)	Elevated Vit.D levels may reduce COVID-19 PCR positivity, D-dimer and CRP levels, as well as the number of affected lung segments
Campi I (2021)[37]	Prospective	103	NA	Low Vit.D levels have an inverse relationship with high IL-6 levels and are independent predictors of COVID-19 severity and mortality
Petrelli F (2021)[39]	Systematic review and meta-analysis (43 observational studies)	612,601	Vit.D deficiency 15, or 20, or 30	Reduced Vit.D is linked to an increased risk of infection, COVID-19 mortality, and severity
Kaya MO (2021)[40]	Systematic review and meta-analysis (21 observational studies)	2,277,860	Low serum Vit.D levels <20, or 50	Vit.D deficiency may increase the risk of COVID-19 infection and the risk of severe disease

Ghasemian R (2021)[41]	Systematic review and meta-analysis (23 observational studies)	11,901	Vit.D sufficiency > 30<br>Vit.D insufficiency 20-30<br>Vit.D deficiency <20	Vit.D deficiency/insufficiency may increase the risk of COVID-19 infection (x3) as well as the risk of severe disease (x5 times)
Hurst EA (2021)[43]	Cross-sectional study	295	Vit.D insufficiency 25-50; deficiency <25 nmol/L	Vit.D deficiency/insufficiency prevalence is high among COVID-19 hospitalised patients
Seal KH (2022)[42]	Retrospective cohort study	4,599	Vit.D deficiency <20	Low Vit.D levels are linked to an increase in COVID-19-related hospitalization and mortality
Israel A (2022)[38]	Observational, retrospective case-control study	2,533	Vit.D >75 nmol/L (normal); 50–75 nmol/L (insufficiency); 30–50 nmol/L (deficiency); < 30 nmol/L (severe deficiency)	Vit.D deficiency is associated to the risk of SARS-CoV-2 infection and severe disease
Pereira M (2022)[26]	Systematic review and meta-analysis (26 observational studies)	8,176	N.A.	Vit.D deficiency is associated to COVID-19 disease severity

COVID-19: Coronavirus Disease 2019; Vit.D: Vitamin D; SARS-CoV-2: Severe Acute Respiratory Syndrome Coronavirus 2; 25(OH)D: 25-hydroxyvitamin D; ARDS: Acute Respiratory Distress Syndrome; ICU: Intensive Care Unit; NA: Not Available; RCT: Randomized Controlled Trial; PCR: Polymerase Chain Reaction; CRP: C-reactive protein; IL-6: Interleukin 6.
